# Transovarial transmission of mosquito-borne viruses: a systematic review

**DOI:** 10.3389/fcimb.2023.1304938

**Published:** 2024-01-03

**Authors:** Sangeeta Janjoter, Divya Kataria, Mahima Yadav, Nisha Dahiya, Neelam Sehrawat

**Affiliations:** Department of Genetics, Maharshi Dayanand University, Rohtak, Haryana, India

**Keywords:** transovarial transmission, *Aedes*, mosquito borne viruses, epidemiology, mosquito defense mechanism, control strategies etc

## Abstract

**Background:**

A number of mosquito-borne viruses (MBVs), such as dengue virus (DENV), zika virus (ZIKV), chikungunya (CHIKV), West Nile virus (WNV), and yellow fever virus (YFV) exert adverse health impacts on the global population. *Aedes aegypti* and *Aedes albopictus* are the prime vectors responsible for the transmission of these viruses. The viruses have acquired a number of routes for successful transmission, including horizontal and vertical transmission. Transovarial transmission is a subset/type of vertical transmission adopted by mosquitoes for the transmission of viruses from females to their offspring through eggs/ovaries. It provides a mechanism for these MBVs to persist and maintain their lineage during adverse climatic conditions of extremely hot and cold temperatures, during the dry season, or in the absence of susceptible vertebrate host when horizontal transmission is not possible.

**Methods:**

The publications discussed in this systematic review were searched for using the PubMed, Scopus, and Web of Science databases, and websites such as those of the World Health Organization (WHO) and the European Centre for Disease Prevention and Control, using the search terms “transovarial transmission” and “mosquito-borne viruses” from 16 May 2023 to 20 September 2023.

**Results:**

A total of 2,391 articles were searched, of which 123 were chosen for full text evaluation, and 60 were then included in the study after screening and removing duplicates.

**Conclusion:**

The present systematic review focuses on understanding the above diseases, their pathogenesis, epidemiology and host–parasite interactions. The factors affecting transovarial transmission, potential implications, mosquito antiviral defense mechanism, and the control strategies for these mosquito-borne viral diseases (MBVDs) are also be included in this review.

## Introduction

Mosquito-borne viruses (MBVs), including dengue (DENV), zika (ZIKV), and chikungunya (CHIKV), are a subset of arboviruses or arthropod-borne viruses that pose a significant risk to both animal and human health due to their worldwide spread (Tingström et al., 2016). MBVs, or moboviruses, have emerged and reemerged as a result of the increased global population growth, urbanization, expanding varieties of mosquito vectors, and easy access to global travel (Peinado et al., 2022). These viruses fall into five genera: *Flavivirus* which includes the dengue, zika, yellow fever, Japanese encephalitis, West Nile viruses (Flaviviridae family), *Alphavirus* which includes chikungunya (Togaviridae family), *Orthobunyavirus*, *Phlebovirus* (Bunyaviridae family), and *Seadornavirus* (Reoviridae family) (Cheng et al., 2016). Due to a lack of therapeutics or effective vaccines, mosquito-borne viral diseases remain a serious global health threat. Mosquito vectors tend to be anthropophilic and are the primary carriers of a number of viruses, including DENV, ZIKV, CHIKV, and yellow fever (Lole et al., 2022). The yellow fever mosquito (*Aedes aegypti*) and the Asian tiger mosquito (*Ae. albopictus*) are the two main vectors of these viruses. Both of the species are extremely invasive, and because of the trade and migration of humans, these are spreading throughout the world. *Ae. albopictus* has a wider geographic range than *Ae. aegypti*, which includes temperate, tropical, and subtropical habitats (Shragai et al., 2017). *Aedes* vectors, primarily *Ae. aegypti*, have drawn special attention due to their rapid spread globally (Lole et al., 2022).

**Figure 1:**
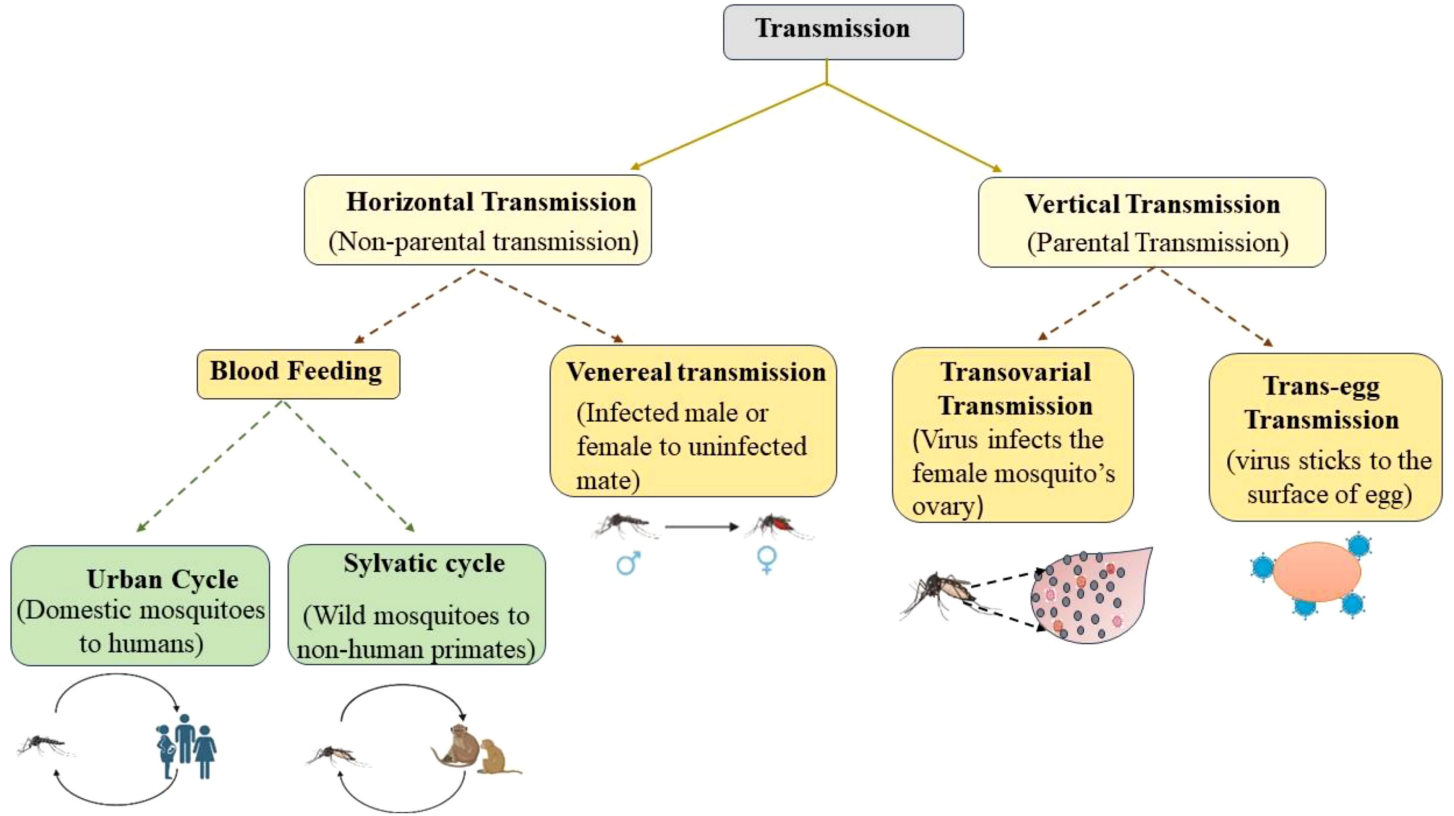
Different modes of transmission of mosquito-borne viruses.

To maturate the fertilized eggs, mosquitoes suck human blood by biting and disseminating the virus between the human host and mosquito vector, parallel to this transmission, the virus can be transmitted directly from parents to their offspring (Yang, 2017). The transmission can therefore be of two types: Vertical Transmission (VT) and Horizontal Transmission (HT) (**Figure 1**). The VT, also known as hereditary transmission, is parental transmission from the female to its offspring, while HT is non-parental transmission, which is mostly preferred by pathogens and includes vector-borne and sexual transmission (Lequime et al., 2016). Blood-feeding invertebrate vectors mainly spread horizontally among the vertebrate host, but they can also do so vertically in the mosquito vector. When the environmental conditions are adverse for HT, VT is thought to be the alternative means for the recurrence of viruses, but its epidemiological importance remains uncertain (Lequime and Lambrechts, 2014). Both in combination are called mixed-mode transmission and is widespread among different taxa of parasites and hosts, including viruses, eukaryotes, and prokaryotes. The use of insecticide spray, the cold and dry seasons in temporal regions and tropics, respectively, can significantly limit vector density and thus, the possibilities for HT. VT provides a crucial link in the transmission cycle when the susceptible host’s density is minimal (Lequime et al., 2016). A thorough scientific study on VT is required to understand the ecology and evolution of these viruses, as epidemiology and infection control mostly depend on the route of transmission. It is possible that the frequent outbreaks of these MBVs may be due to their remarkable capacity for VT and their survival for longer periods of time. The number of females that transmit these viruses to their offspring vertically is the rate of VT, depending on a variety of determining factors, such as virus strain, mosquito species, gonotrophic cycle, blood meal, and climate, of which temperature has a significant impact, that is, the lower the temperature, the higher the infection rate. Mosquito age also affects the rate of VT in that older mosquitoes have a reduced capacity to transmit viruses (Kaavya et al., 2022). Larvae are more susceptible to infection than adult mosquitoes and this is because adult mosquitoes develop a peritrophic matrix immediately after blood feeding; therefore, viruses can cause infection only while mosquitoes are in larvae form. They typically directly infect the midgut, which increases the risk of infection (Lutomiah et al., 2007).

**Figure 2:**
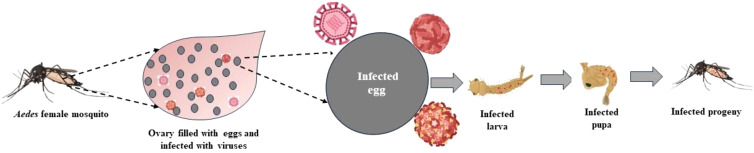
Pathway of transovarial transmission.

Transovarial Transmission (TOT) and Trans-egg Transmission (TET) are the two ways of VT through which these viruses spread. In TOT, the female germinal tissues are infected by the virus, including the oocytes, during the development stage, that is, before the fertilization of the eggs (as shown in **Figure 2**). Trans-ovum, or TET, occurs during fertilization or during oviposition, when the egg surface is contaminated by the virus, or it could be said that the virus adheres to the surface of the egg (Sánchez-Vargas et al., 2018; Kirstein et al., 2022). It is possible that these two transmission mechanisms coexist in *Aedes* vectors at the same time (Lai et al., 2020). TOT is a type of VT in which the infected female mosquitoes pass on the virus to their progenies via ovaries when their eggs are laid. When environmental conditions are not favorable for mosquito development, such as during winter and dry seasons in temperate and tropical areas, TOT is believed to be the crucial mechanism for the persistence of the virus (Heath et al., 2020). Therefore, vertical transmission is the strategy for MBV survival in nature during adverse climatic conditions (Thangamani et al., 2016). It takes time for viruses to enter the bloodmeal and to integrate into the ovaries; therefore, transmission occurs only after several weeks gonotrophic cycles (Agarwal et al., 2014). Numerous studies demonstrate that after infection, the virus spreads through the hemolymph to the hemocoel and secondary tissues, including the ovaries, and for the virus to persist in the ovaries, it must endure stressful circumstances during the development of the host mosquito, that is, when it is in the larval instar, pupal, and adult stages (Agarwal et al., 2014; Kaavya et al., 2022). Not all MBVs prefer TOT; *Flaviviruses*, for example, favor both TOT and TET. TOT is significantly more advantageous and promising than TET and also provides more chances of viral infection in the subsequent generation (Kaavya et al., 2022). To evaluate whether TOT has taken place or not, immunocytochemical methods can be used (Kurnia et al., 2022). Numerous studies have been conducted on TOT in *Aedes*, with a particular emphasis on dengue and chikungunya viruses (Li et al., 2017). Cytoplasmic inheritance, which has been best understood through research on cell organelles such as mitochondria, chloroplasts, and intracellular organisms, is the primary mechanism for transovarial transmission. These cytoplasmic factors are transferred to the daughter cells in a specific sequence at the time of meiosis, and the intracellular symbionts are also successfully transmitted through this mechanism. The mechanism of TOT in mosquitos, however, is less understood (Smith and Dunn, 1991). The virus must infect mosquito germline in order for transmission to be successful. The molecular interaction begins with the attachment of virus particle to its target via receptor i.e. binding of viral surface molecule to cellular receptor of mosquito. The interaction between virus and its receptor is highly specific and, yet a virus family may share a common receptor. For the entry of virus into the target cell, lipid bilayer of cell membrane fuses with the enveloped virus and receptor-mediated endocytosis takes place for non-enveloped viruses and from there the viral genome enters into the cell cytoplasm for further transmission (Dimmock et al., 2015). 

## Data sources and search strategy

We used the PubMed, Scopus, and Web of Science databases to search for the publications discussed in this systematic review, and also the references included in relevant articles. The information about mosquito-borne viruses, diseases and their symptoms, outbreaks, and cases was gathered from the websites such as World Health Organization (WHO) (https://www.who.int/news-room/fact-sheets) and the European Centre for Disease Prevention and Control (https://www.ecdc.europa.eu/en/chikungunya-monthly, 2023).

The full-text versions of some papers were not available on PubMed, and so these were obtained from ResearchGate and through requests sent to the corresponding authors via email. The search terms “transovarial transmission”, “transmission”, “mosquito-borne viruses”, “dengue”, and “mosquito immunity” were used in different combinations when searching for publications to be discussed in this study. For each research and review article the title and abstract were scrutinized separately. The full text of only the articles with titles and abstracts that matched our topic was read. The study includes the reviews and research publications that were relevant to the subject. The most recent literature search was completed on 20 September 2023. We included only those articles in our study that describe the vertical transmission and transovarial transmission of DENV, ZIKV, and CHIKV; the articles describing horizontal transmission and viruses other than these were excluded. The references used were taken mainly from the papers published after the year 2000, with the exception of a few.

## Results

**Figure 3:**
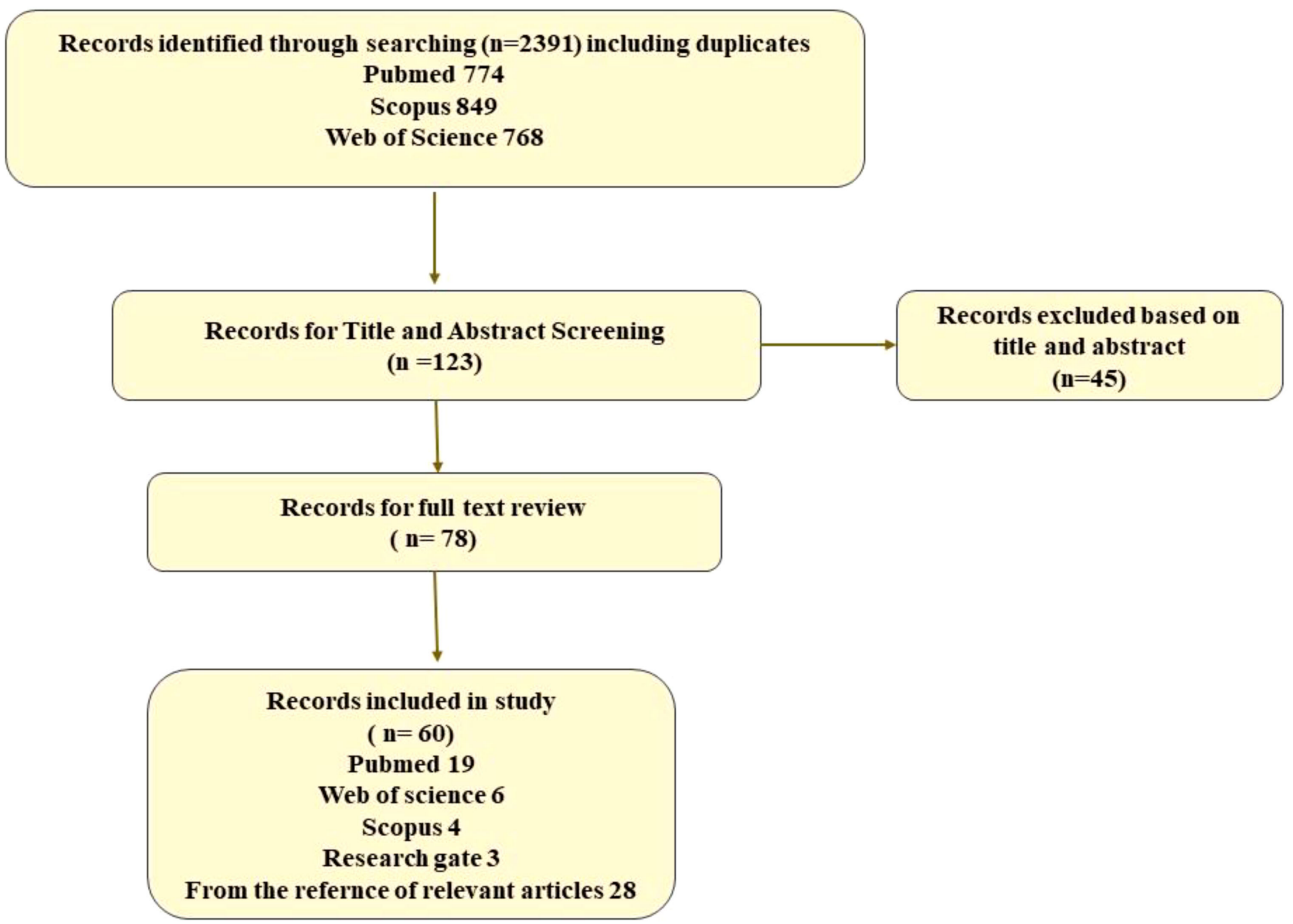
A flow diagram of the literature search.

A total of 2,391 articles were found through the manual and electronic searches, and after eliminating duplicates and evaluating the titles and abstracts, 123 studies were selected for full-text review. After further screening, 60 articles were included in this systematic review (**Figure 3**).

### Zika virus

ZIKV, belong to the Flavivirdae family, contains single-stranded and positive-sense RNA and causes zika disease (Dahiya et al., 2022). *Ae. aegypti* is the primary vector for this disease. ZIKV has two cycles: the first is the sylvatic cycle (occurring between non-human primates and wild-type mosquitoes) and the second is the urban cycle (occurring between local mosquitoes and humans). Headache, fever, joint pain, meningoencephalitis, myelitis, myalgia, maculopapular rash, and microcephaly are the major symptoms of ZIKV. (Dahiya et al., 2023; Aliota et al., 2016a). As reported by Kendra et al., following ZIKV infection, the virus multiplies and penetrates the placental barrier during pregnancy. ZIKV causes fetal microcephaly through vertical transmission (Quicke et al., 2016),. ZIKV is well known throughout the world due to the fatal neurological disorders it can cause, including Guillain–Barré syndrome (Lai et al., 2020). Although 80% of patients are asymptomatic, only a small proportion show clinical symptoms (Blagrove et al., 2020). It has been confirmed experimentally that *Ae. aegypti* is a highly efficient and competent vector of ZIKV. However, there is evidence that different species of mosquito have different competencies as vectors of this virus. The first strain (MR766) of zika virus was isolated from rhesus macaque monkey from the zika forest of Uganda in 1947, and in 1950 the first human infection by zika was reported in Africa (Ezzati-Tabrizi et al., 2013). Throughout Asia and Africa, cases of zika were frequently detected from 1960 to 1980, and from February 2016 to November 2016, zika-related microcephaly was declared a public health emergency of international concern by the WHO (WHO, 2022a). In India, the zika virus was first reported in 2017, and reemerged in 2021. As of December 2022, zika virus has been reported in 89 countries and territories (WHO, 2022a). Studies have confirmed that ZIKV can be transmitted by *Ae. albopictus* and *Ae. aegypti* via eggs, that is, through TOT (Li et al., 2017). An immunohistochemistry (IHC) study provides the first indication of ZIKV location, demonstrating that TOT is more effective than TET. The germarium gets infected when ZIKV reaches the ovary and a new follicle emerges from the infected germarium and grows to maturity in each consecutive gonotrophic cycle, which causes the virus to replicate within the developing embryo and be vertically transmitted. A severe infection of ZIKV has no pathological effect on the ovaries, oviposition, or ovarian development, but has a deleterious impact on oogenesis, which means it reduces the number of eggs formed in the ovaries (Lai et al., 2020).

### Dengue virus

Dengue viruses are positive-sense, ssRNA *Flaviviruses* ~11 kb in length that belong to the family Flaviviridae (Joshi et al., 2002). Female *Ae. aegypti* mosquitoes are the main dengue-transmitting vectors, and the disease is caused by four antigenically diverse serotypes (DENV-1-DENV-4) (Guedes et al., 2010) that share a common epidemiology and a comparable pattern of disease in humans (Gubler, 2002). Dengue viruses are globally endemic and spread in tropical and subtropical regions (Gubler, 2002). The patient may or may not show symptoms; in the case of symptomatic dengue, severe fever (up to 104°C), headache, nausea, rash, pain, aches, and leucopenia can be seen. It is very likely that the persistence of the dengue virus is owing to cases of asymptomatic illness and travel by humans (Grunnill and Boots, 2016). The first ever dengue transmission was reported in Afghanistan, but local transmission was first seen in France and Croatia in 2010. Currently, the disease is endemic in approximately 100 countries in the WHO region of almost every continent. Asia accounts for ~70% of the global disease burden, and according to an estimate, an average of 390 million dengue cases have been reported globally every year, with the largest number of cases reported in 2019. Dengue continues to affect Brazil, Colombia, Cook Islands, Fiji, India, Kenya, Paraguay, Peru, the Philippines, Réunion, and Vietnam as of 2021 (WHO, 2022a). In 1983, Rosen et al. demonstrated the TOT in all four dengue virus serotypes in *Ae. albopictus* vectors and found that the rate of transmission in all serotypes was different, with DENV-1 generally having the highest rate, and DENV-3 having the lowest rate (Rosen et al., 1983). *Ae. albopictus* has a higher transmission rate for DENV than has *Ae. aegypti* (Lequime et al., 2016). According to a study, it has been shown that dengue virus can be orally spread by transovarially infected female mosquitoes. Their study also revealed that after the incubation of several, when eggs are obtained from infected mosquitoes, the rate of vertical transmission increases (Joshi et al., 2002). The detection of DENV in field-collected mosquito larvae and adult male mosquitoes indicates that males may contribute to the persistence of DENV in nature (Sánchez-Vargas et al., 2018). The cause of DENV outbreaks is the TOT because of the endurance of the virus between the period of epidemics (Saepudin et al., 2022). Kurnia et al. have demonstrated the TOT in *Ae. aegypti and Ae. albopictus* (Kurnia et al., 2022). A relatively lower rate of TOT has been found in *Ae. aegypti* (Yang, 2017). It has been proposed that TOT may have a potential impact on the epidemiology of dengue, and it is considered a key mechanism for the persistency of the virus in the vector during inter-epidemics. Studies have revealed that both climatic variables, such as temperature, humidity, and rainfall, and non-climatic variables, such as herd immunity, have an influence on the dynamics of DENV transmission, and that climatic factors are the key predictor of dengue outbreaks (Thongrungkiat et al., 2011).

### Chikungunya

CHIKV is an *Alphavirus* that belongs to the Togaviridiae family and causes chikungunya, a mosquito-borne infectious disease. Its viral genome consists of approximately 11.8 kb long, linear, positive-sense, single-stranded RNA molecules. The illness is transmitted through the bite of infected *Aedes* mosquitoes (Sudeep and Parashar, 2008). Approximately 85% of patients exhibit symptoms such as high fever, acute joint pain, severe arthralagia, rash, headache, and photophobia, and 30%–40% of infected individuals experience chronic joint illness that can last for weeks, months, or years after the initial infection (Shragai et al., 2017). The first CHIKV epidemics was originally recorded between 1952 and 1953 in Tanzania. The first instance in Asia occurred in Bangkok in 1958. In India the first case was found in Kolkata in 1963, and CHIKV reappeared in 2005 (Sudeep and Parashar, 2008). It has now been reported in approximately 110 countries all over the world (WHO, 2022b). According to a recent report by the European Centre for Disease Prevention and Control (ECDC), as of 23 August 2023, 3,20,000 cases and 340 deaths were reported worldwide, with the largest number of (2,09,489) cases reported in Brazil and the largest number of deaths (271) reported in Paraguay (ECDC, 2023). According to Mourya’s 1987 report, there is no evidence of the TOT of CHIKV by *Ae. albopictus*. Zytoon tested the possibility of TOT using CHIKV (African strain), *Ae. albopictus* (Miki strain), and microfilariae to evaluate the feasibility of the TOT of the virus and their findings showed that *Ae. albopictus* has a limited ability to spread CHIKV through TOT (Zytoon et al., 1993). The large number of CHIKV outbreaks in Réunion throughout 2005, in the Kerala state of India and in Italy in 2007, in the Guangdong province of China in 2010, and in France between 2010 and 2014 is due to the mutation of the CHIKV envelope protein E1 (E1-A226V), a mutant virus strain that can spread throughout mosquito populations via TOT because male offspring can pass the virus through mating to females that are uninfected (Wang et al., 2022). The mechanism that causes CHIKV to be more prevalent during unfavorable times, particularly in the winter, however, is unknown (Agarwal et al., 2014).

## Factors affecting transmission

The importance of the interaction between the vector and virus for effective TOT has been demonstrated by numerous studies, which tend to suggest that TOT is the consequence of convergent evolution and that environmental ecology puts immense stress on viruses to evolve to facilitate TOT (Bergren and Kading, 2018). The central approach for managing arbovirus disease is vector control measures, hence there is a need to understand how arboviruses infect competent mosquito vectors and develop disease, various factors that affect the viral transmission in the mosquito vector and probable mechanism to escape antiviral barriers. There are two types of factors affecting viral transmission namely intrinsic and extrinsic factors. Intrinsic factors include those affecting the transmission process from within the body, such as midgut escape barrier (MEB), midgut infection barrier (MIB), salivary gland infection barrier (SGIB), salivary gland escape barrier (SGEB) (Franz et al., 2015). Extrinsic factors include temperature, humidity, rainfall etc. Mosquitoes are poikilotherms and their body temperature fluctuates significantly with fluctuation in the surrounding environment and the biological traits of mosquitoes, such as their fecundity and survival rate, in addition to their interactions with mosquito-borne viruses, are greatly influenced by temperature (Shragai et al., 2017; Jian et al., 2023). When the external temperature is low, the virus may not have sufficient time to replicate, penetrate the salivary gland, and become pathogenic within the vector’s lifespan, which indicates that an appropriate threshold temperature is critical for TOT (Blagrove et al., 2020). Carrington reported that there is a positive correlation between temperature and fertility in *Ae. albopictus*, that is, the number of eggs and the transmission rate increase as the temperature rises (Carrington et al., 2013). Limited virus invasion and lower infection rate are caused by a low temperature, which lessens the virus’s capacity to penetrate the mosquito midgut barrier (Jian et al., 2023). These viruses persist throughout the year, while the highest peaks for infections caused by mosquito populations tend to correspond with periods of higher rainfall and humidity conditions (Heath et al., 2020). It is commonly seen that in densely populated areas, the rate of virus transmission is usually higher than in areas with fewer human inhabitants. In addition, the nutrition obtained by the viruses can be a determining factor for their successful transmission (Kramer and Ciota, 2015).

## Mosquito antiviral defense mechanism

**Figure 4:**
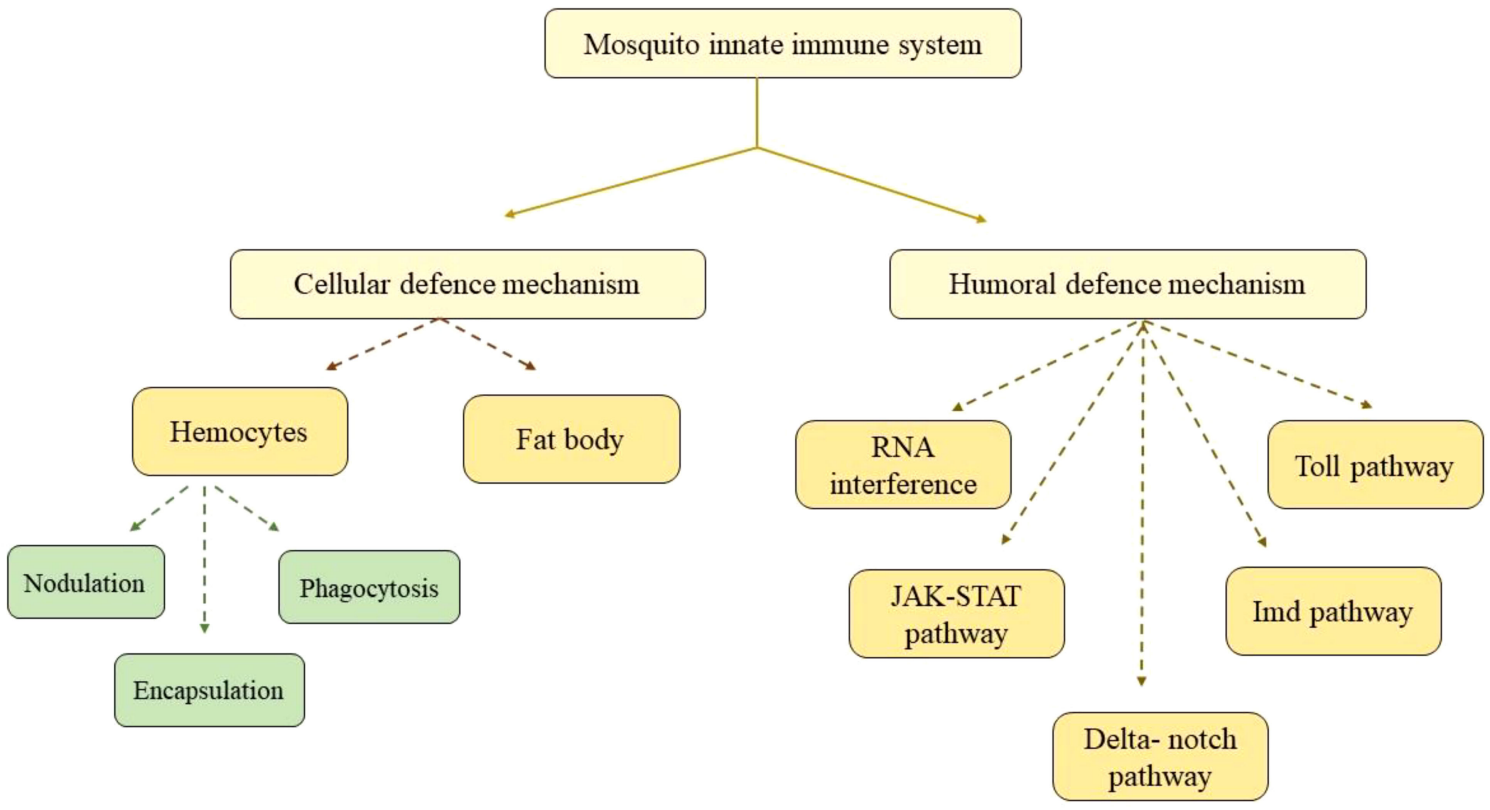
Flow chart showing various mechanisms of the mosquito innate immune system.

In the last 10 years, comprehensive genome sequencing and annotation have greatly contributed to the rapid advancement of our understanding of the mosquito immune system (Cheng et al., 2016). After a mosquito ingests the virus-infected blood meal from an infected host, the virus enters its midgut epithelium and then travels to the hemocoel and then to secondary organs, including the salivary gland (Franz et al., 2015). For the virus to be transmitted to its subsequent host, it needs to travel to the salivary gland (Chauhan et al., 2012). Mosquitoes have only innate immune system as their defense machinery, which is activated by viral infection, causing the antiviral response-related gene to be transcribed (Lee et al., 2019). The immune system of mosquitoes has two correlated responses: cellular and humoral defense responses. These two act together to defend the mosquito from a wide range of pathogens (Satyavathi et al., 2014). Various pathways for the mosquito immune system are schematically shown in **Figure 4**.

### RNA interference pathway

RNA interference (RNAi) is the central antiviral mechanism in insects, particularly in controlling virus infection through the degradation of RNA, also known as RNA silencing (Lee et al., 2019). The major event of this pathway is the degradation of long viral double-stranded RNA into small RNAs. The small RNAs are of three types (i) small interfering RNA (siRNA), (ii) microRNA (miRNA), and (iii) PIWI-interacting RNA (piRNA) (Cheng et al., 2016). When the mosquito genome replicates, it produces an intermediate double-stranded RNA, which binds to the Dicer-2-R2D2 complex. This complex comprises a RNase III enzyme (Dicer-2) and a protein attached to it (R2D2); Dicer-2 cleaves the dsRNA and produces siRNA (21–23 nucleotides). Subsequently, siRNA further binds to the RNA-induced silencing complex (RISC), and one of the strands of dsRNA is degraded. The RNA then becomes single stranded and act as a guide strand for the detection and degradation of the viral RNA with the help of argonaute-2 in a specific sequence. (Donald et al., 2012). If the RNAi pathway is impaired or silenced, the viral replication and dissemination during DENV-2 infection increases, indicating its significance as an antiviral defense mechanism (Lee et al., 2019). By silencing the genes linked to fertility, behavior pattern, survival, and vector status in mosquitoes, RNAi offered fresh insights into fundamental research that may be used to lessen the burden of diseases spread to humans by mosquitoes (Yadav et al., 2023).

### JAK-STAT pathway

The mosquito has cytokine receptor Domeless (*Dome*) and tyrosine kinase Hopscotch (*Hop*), these act together and activate the JAK-STAT pathway (Souza-Neto et al., 2009). In this pathway, the ligand binds to the dome and a conformational shift occurs, which leads to the self-phosphorylation of *Hop* (JAK). The activated *Hop* then phosphorylates the Dome with the formation of a docking site for cytosolic STAT. STAT then binds to the Dome–Hop complex, which undergoes phosphorylation. The phosphorylated STAT first dimerizes and then moves to the nucleus; this activates the transcription of specific effector genes with antiviral immune roles (Souza-Neto et al., 2009; Lee et al., 2019). Activating JAK-STAT pathway, before or just after the DENV infection could severely limit viral replication, possibly to the point where DENV transmission would be negatively impacted. Functional and reverse genetic studies show that JAK-STAT pathway enhances resistance to DENV serotypes (DENV2 and DENV4) in *Ae. aegypti* but, when it came to two other significant arboviral diseases, chikungunya and zika, the pathway activation did not give resistance. Functional and reverse genetic studies show that the JAK-STAT pathway enhances resistance to DENV and ZIKV in *Ae. aegypti* (Angleró-Rodríguez et al., 2017). Most studies on the JAK-STAT pathway in mosquito immunity have focused on dengue infection, but pathway activation in response to other viruses and the downstream process may vary for each virus. The overexpression of *Hop* by transgenic bacteria in the gut was observed to reduce the severity of DENV infection instantly, but for ZIKV, the reduction in infection severity was seen on day 7, and for CHIKV, this pathway was not at all effective (Jupatanakul et al., 2017).

### DELTA–NOTCH signaling pathway

This pathway is important for stem cell maintenance, embryonic development, and adult tissue renewal, and it also reduces the replication of DENV in *Ae. aegypti* (Kopan and Ilagan, 2009). The signaling is initiated when the delta ligand binds to the notch transmembrane receptor and proteolysis occurs. After this, an active fragment called the notch intracellular domain, which activates the downstream signaling genes in the nucleus, is released (Lee et al., 2019). During DENV infection, the components of this pathway are upregulated in *Ae. aegypti* mosquitoes (Serrato-Salas et al., 2018). The activation of this signaling system results in endoreplication, a process in which cells perform several rounds of DNA replication without mitosis to greatly increase the genomic DNA content of the cells. Endoreplication induction enhanced the number of gene transcripts involved in viral spread control (Serrato-Salas et al., 2018; Lee et al., 2019).

### TOLL and IMD pathway

Pathogens activate both the TOLL and IMD pathways by binding pathogen-associated molecular patterns (PAMPs) to the host’s pattern recognition receptor (PRRs), resulting in a cascade of events that engage the immune AMP producing effector genes (Yu et al., 2020). The cytokine Spatzle (Spz), a ligand that binds to the Toll transmembrane receptor, is cleaved to begin the Toll pathway. Activated Toll initiates signaling via the Toll-associated adaptor proteins MyD88, Tube, and Pelle kinase. Cactus, the antagonist of the Toll pathway, is then phosphorylated and undergoes proteasomal degradation, which results in the movement of the transcription factor Relish 1 from the cytoplasm to the nucleus, binding to kB motifs on the promoters of several AMPs genes, including *Diptericin* and *Cecropin*, which are effective against fungi and Gram-positive bacteria (Lee et al., 2019; Yu et al., 2020). When the IMD pathway is activated, Caspar, a negative regulator, is degraded. This causes Relish 2 (Rel2) to be translocated to the nucleus, where it triggers the transcription of AMPs (Xi et al., 2008). During the DENV infection of *Ae. aegypti*, the expression of the Toll pathway genes (*GNBP*, *Toll5A*, and *MYD88*) was elevated in the salivary gland. The silencing of *MYD88* resulted in a modest rise in the severity of DENV infection in the intestine (Luplertlop et al., 2011).

## Cell-mediated immune response

Hemocytes are cells that circulate inside the hemolymph and are susceptible to viral infections such as DENV, SINV, and WNV (Lee et al., 2019). The hemocyte-mediated immune response is rapid and includes pattern recognition; phagocytosis (haemocyte cells recognize the intruding pathogen, engulf, and destroy it intracellularly) (Lamprou et al., 2007); nodulation (a large number of microbes is entrapped by the accumulation of haemocytes) (Satyavathi et al., 2014); melanization (production of melanin for the encapsulation of invading pathogens); and the synthesis of antimicrobial peptides and initiation of signaling cascades for cytotoxic effectors to eradicate infection (Christensen et al., 2005).

The fat body of an insect is an organ that acts similarly to adipocytes and liver in mammals. According to one recent study, the JAK-STAT pathway is activated in the fat body of *Ae. aegypti* during dengue virus infection, but this pattern was not observed for CHIKV and ZIKV. This means that different viruses have different responses to the JAK-STAT pathway (Jupatanakul et al., 2017). Although insect antiviral immune responses are triggered during viral infections, viruses are not entirely eradicated from the mosquitoes. Instead, a persistent infection develops in mosquitoes, making them effective carriers for viral illnesses at little to no cost to the host. However, little is known about how viruses persistently infect mosquitoes. Recent research has shown that virus-derived DNA (vDNA) produced during viral infection is crucial for mosquito survival and chronic infection (Poirier et al., 2018).

The majority of viruses spread by mosquitoes are RNA viruses, and after infection, the host cell’s reverse transcriptase converts viral RNA, or truncated copies of the viral genome, known as defective viral genomes (DVGs), into vDNA, to stop viral replication. The vDNAs then activate the RNAi apparatus. When exposed to a cognate virus, vDNA is sufficient to create siRNAs that cause an antiviral response. In addition, suppressing vDNA production makes individuals incredibly vulnerable to viral infections (Poirier et al., 2018; Lee et al., 2019).

## Control strategies

The use of mosquito nets and insecticides to reduce human–vector interaction is among the vector control strategies that are largely used in preventing and treating the diseases spread by mosquitoes (Lee et al., 2019). In spite of these traditional methods, there is a need for new interventions to control the transmission of vector-borne diseases. The Vector Control Advisory Group (VCAG) assist the WHO in creating public health policy on new tools and techniques. Insecticide-treated nets (ITNs) that can kill or sterilize mosquitoes, such as the pyrethroid–piperonyl butoxide net and the pyrethroid–chlorfenapyr net, can be used, in addition to spatial repellents (volatile chemicals), and vector traps such as adulticidal oviposition traps that attract adult mosquitoes to lay their eggs in traps and larvicidal traps to kill their larvae (WHO VCAG). Larval control, specifically through the use of microbial and chemical larvicides, is the primary way of viral outbreak control due to mosquitos, particularly *Ae. aegypti* (Achee et al., 2019). The two innovative vector control methods currently in use are genetically modified and non-genetically modified. Genetic strategies targeting Aedes vector are the most advanced methods for the prevention of dengue and chikungunya transmission. Genetically modified strategies include the Release of insects carrying dominant lethal (RIDL) genes and gene drive system. In RIDL technology, dominantly inherited lethal gene is artificially inserted into the genome of mosquito, causing 97% death of heterozygous F1 progeny. CRISPR-Cas9 technology is used in the gene drive strategies in order to increase the frequency of desired traits in the succeeding generations (WHO VCAG). The genomes of various mosquito species such as An. stephensi, Ae. aegypti, Cx. quinquefasciatus, and *Cx.* pipiens have recently been modified using the effective technology of CRISPR/Cas9-based gene editing (Lee et al., 2019). Nongenetically modified strategies include Sterile Insect Technique (SIT) and Wolbachia-centred biopesticide strategies. SIT has been effectively employed to control a variety of disease vectors for many years. In this technique, males of target species are previously sterilized by irradiation treatment and then released to fight with wild males for females to breed. This technique have the ability to decrease the wild population and possibly result in localized eradication if they are repeated over an extended period of time (Lee et al., 2019). With the aim of lowering the reproductive success of and viral capacity in the mosquito population, approaches such as releasing *Wolbachia*-infected mosquitoes and genetically engineered mosquitoes into native mosquito populations have been used over the past 10 years (Lee et al., 2019). Due to a lack of approved vaccines and efficient antiviral treatments, however, viruses have been persistently spreading. To combat this problem, the intracellular bacterium, *Wolbachia*, (inherited maternally and present in various insect species such as mosquitoes, ants, bees, butterflies, and beetles) can be introduced in *Ae. aegypti* mosquitoes for arbovirus control and prevention. In fact, *Wolbachia* biocontrol could be utilized against the four viruses discussed in this study, namely CHIKV, DENV, YFV, and now ZIKV; however, it has not been determined if ZIKV can infect, spread, and be transmitted by *Wolbachia*-infected *Ae. aegypti* (Aliota et al., 2016a). *Wolbachia* strains of fruit fly in *Ae. aegypti* mosquitoes can shorten the lifespan of mosquitoes, interfere with mosquito reproduction, and can affect the replication of viruses through invasion (Achee et al., 2019).Some strains of *Wolbachia* bacteria reduce the replication virus and mosquito lifespan concurrently (Aliota et al., 2016b). The strains *wMel* and *wMelPop* from *Drosophila melanogaster* and *wAlbB* from *Ae. albopictus* strains of *Wolbachia* have been used in *Ae. aegypti*; of these, *wMel* has the potential to reduce the vectorial capacity of *Ae. aegypti* for both DENV and CHIKV, and favorable results have also been found recently in ZIKV (Achee et al., 2019). New mosquito strategies may be made possible by understanding the typical viruses and gut microbes that invade reproductive organs and go through VT (Kaavya et al., 2022). The genomes of various mosquito species, such as *Anopheles stephensi*, *Ae. aegypti*, *Culex quinquefasciatus*, and *Culex pipiens* have recently been modified using the effective technology of gene editing based on CRISPR–Cas9 (Lee et al., 2019).

## Conclusions

MBVs represent a serious public health threat worldwide due to their global prevalence. They can be transmitted vertically in mosquito vectors via transovarial transmission, that is, through the ovaries, in order to survive in adverse conditions. Many studies have been conducted on TOT in *Aedes* with particular emphasis on DENV and CHIKV. The transmission gets established during the second gonotrophic cycle, and it eventually declines after further transmission. We studied the factors influencing the transmission including temperature, humidity, midgut and salivary gland escape and infection barriers. Mosquito have some antiviral cellular and humoral defense mechanisms through which it can protect itself from the viral infection. Along with the traditional insecticide treated nets and chemical methods of vector control, there is a need to extend this approach to new techniques of gentic and non-genetic modification of vectors for their management. The development of novel strategies to eradicate diseases spread by mosquitoes was made possible by the employment of gut bacteria particularly *Wolbachia* in paratransgenic approach. Although work is being done for these new strategies there is not sufficient eradication of diseases caused by these vectors. The current study enhances the understanding of transovarial transmission of *Alphavirus* and *Flavivirus* in *Aedes* vectors and also sheds light on the factors affecting transmission and vector control strategies. Additional research is needed to understand the molecular mechanism of TOT. In addition, if we are to learn more about viral prevalence and stability in challenging environmental circumstances, more research is required.

## Author contributions

SJ: Conceptualization, Investigation, Visualization, Writing – original draft, Writing – review & editing. DK: Conceptualization, Investigation, Visualization, Writing – original draft, Writing – review & editing. MY: Resources, Writing – review & editing. ND: Resources, Writing – review & editing. NS: Conceptualization, Supervision, Writing – review & editing.
